# Global preventive feedback of powerful radio jets on galaxy formation

**DOI:** 10.1073/pnas.2402435121

**Published:** 2024-08-19

**Authors:** Renyue Cen

**Affiliations:** ^a^Center for Cosmology and Computational Astrophysics, Institute for Advanced Study in Physics, Zhejiang University, Hangzhou 310027, China; ^b^Institute of Astronomy, School of Physics, Zhejiang University, Hangzhou 310027, China

**Keywords:** galaxy formation, intergalactic medium, magnetic field, AGN feedback, radio jets

## Abstract

Firmly anchored on observational data, giant radio lobes from massive galaxies hosting supermassive black holes can exert a major negative feedback effect, by endowing the intergalactic gas with significant magnetic pressure hence retarding or preventing gas accretion onto less massive halos in the vicinity. Since massive galaxies that are largely responsible for producing the giant radio lobes, this effect is expected to be stronger in more overdense large-scale environments, such as protoclusters, than in underdense regions, such as voids. We show that by redshift z=2 halos with masses up to $\left(10^{11\;\;\text{to}\;12},10^{12\;\;\text{to}\;13}\right)\;M_{⊙}$ are significantly hindered from accreting gas due to this effect for radio bubble volume filling fraction of (1.0,0.2), respectively. Since the vast majority of the stars in the universe at z< 2 to 3 form precisely in those halos, this negative feedback process is likely one major culprit for causing the global downturn in star formation in the universe. It also provides a natural explanation for the rather sudden flattening of the slope of the galaxy rest-frame UV luminosity function around z∼2. A cross-correlation between protoclusters and Faraday rotation measures may test the predicted magnetic field. Inclusion of this external feedback process in the next generation of cosmological simulations may be imperative.

## Significance

Negative feedback processes from the growth of supermassive black holes are believed to play a central role in the formation of galaxies. Internal feedback processes by essentially driving gas away from galaxies have been widely used in cosmological simulations. In this article, we investigate a distinct type of negative feedback process, termed external negative feedback, due to powerful radio jets by energizing intergalactic medium. The intergalactic medium endowed with significant magnetic fields is retarded or prevented from entering subsequent halos. This external feedback process is in a sense proactive and preventative, in contrast to the case of interval feedback processes. Inclusion of this external feedback process in the next generation of cosmological simulations may be imperative.

Cosmological hydrodynamic simulations have entered its fourth decade since the pioneering works in the late eighties and early nineties of the last century (e.g., refs. 1, 2, 3, 4). In the first two decades, the focus was largely on the evolution of the intergalactic medium, regions significantly removed from star formation in galaxies, with a number of notable successes, including the finding of the fluctuating nonlinear Gunn–Peterson cosmological density field as the origin of the Lyman alpha forest (5–8), a successful account of the missing baryons in the universe at zero redshift (9, 10) and the discovery of two modes of gas accretion into galaxies (11). In the most recent two decades, driven in part by the availability of large computing power, we witnessed large-scale cosmological simulations with increasingly high numerical resolutions and ever more sophisticated implementations for feedback processes from stellar evolution and growth of supermassive black holes (e.g., refs. 12, 13, 14, 15, 16, 17, 18, 19, 20, 21, 22, 23). On the feedback processes from AGN, the mainstream implementations of various modes (QSO or radio mode) can be classified as internal feedback processes by pushing entered gas away from galaxies.

In the article, we put forth an important AGN energy source, namely, the powerful Fanaroff-Riley (FR) II radio jets, for regulating the thermodynamic state of the cosmic gas, especially in the low-density intergalactic medium (IGM). FR II jets are observed to transport a large amount of energy to megaparsec scale, deep into the low-density IGM (e.g., ref. 24). Preferentially affecting low-density gas is economical energetically by maximizing entropy generation. In contrast to internal feedback processes currently employed in cosmological simulations, this distinct feedback process is of global, collective, and multigenerational nature, where radio lobes generated by earlier galaxies will retard, reduce, or prevent subsequent gas accretion onto galaxies in the neighborhood.

## Suppression of Galactic Gas Accretion Due to Intergalactic Magnetic Field

We consider the simplest case that powerful AGN radio jets send magnetic fields permeating the entire universe but will discuss later if filling fraction is less than unity, the likely case. Let us denote the mean magnetic field of the universe due to AGN jets as $B_{i}$ at certain redshift. Then under the adiabatic assumption, when a small gas parcel is eventually brought to the virial surface of a halo with a density equal to the virial overdensity $\delta_{v}$, the magnetic field would have increased to $B_{v}$,

[1] $$\begin{array}{c} B_{v}=B_{i}\delta_{v}^{2/3}. \end{array}$$

Can this gas parcel enter this virial radius? To answer this question, let us make a most conservative assumption that this gas has completely lost its thermal pressure when arriving at the virial surface of the halo. Under a likely valid assumption that it is not self-gravitating (because otherwise it would have entered a halo already), the magnetic pressure force is dynamically analogous to thermal pressure force in terms of retarding the gas from accreting onto the halo. When the magnetic pressure force exceeds gravitational force due to the dark matter halo at the virial radius, or equivalently, the magnetic temperature, defined as $T_{B}\equiv B_{v}^{2}/8\pi n_{v}k_{B}$, where $n_{v}$ is gas number density at the virial radius and $k_{B}$ the Boltzmann’s constant, exceeds the virial temperature of the halo, the gas parcel will be prevented from entering the halo. This statement can be expressed in terms of a threshold Alfv$\overset{´}{\;\text{e}}$n velocity $v_{A}=\sqrt{B_{v}^{2}/4\pi \rho_{v}}$ (where $\rho_{v}=m_{p}n_{v}$)

[2] $$\begin{array}{c} \begin{array}{c} v_{A}=\sqrt{2}\sigma_{v}, \end{array} \end{array}$$

where $\sigma_{v}$ is the 1-d velocity dispersion of the halo.

**Fig. 1.**
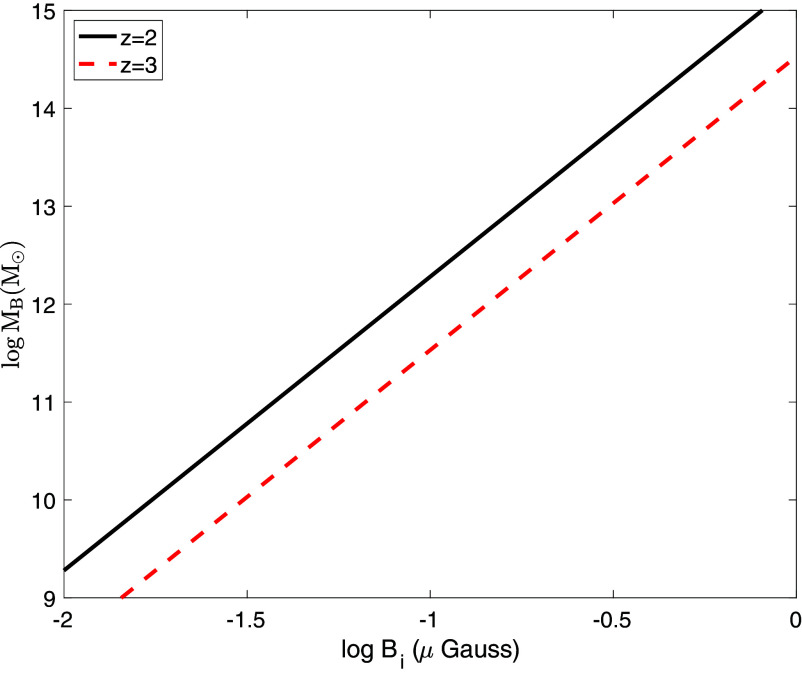
shows the magnetic Jeans mass, $M_{B}$, as a function of the magnetic field in the mean density of the universe, $B_{i}$, at z=2 (black solid curve) and z=3 (red dashed curve).

Combining Eqs. **1** and **2**, along with the relation between halo mass and $\sigma_{v}$, we obtain the results shown in Fig. 1. A more precise formulation may be quantified with detailed simulations, in a way similar to the Jeans filtering effect demonstrated insightfully in ref. 25 and is reserved for a future study. Nevertheless, we see that, if an initial mean field $B_{i}$ of order 0.1 μG is reached, gas accretion onto halos as massive as $M_{h}∼10^{12}\;M_{⊙}$ will be significantly affected. In the next section, we examine whether such a field may be injected into the intergalactic medium by powerful radio jets.

## Intergalactic Magnetic Field Due to Powerful Radio Jets

To estimate the energetics of powerful radio jets, we introduce a few variables. We denote the global ratio of supermassive black hole (SMBH) mass to stellar mass as β adopting 0.002 measured locally (e.g., ref. 26), although there is preliminary evidence that this ratio may increase with increasing redshift (e.g., ref. 27). We denote the fraction of SMBH rest mass energy ($M_{\mathrm{SMBH}}c^{2}$) released in the form of powerful radio jets as $\eta_{J}$ for the radio loud galaxy population, and $\eta_{R}$ as the fraction of all relevant galaxies classified as radio loud galaxies. To obtain the fraction of jet energy in the form of magnetic energy, $\eta_{B}$, we have performed magnetohydrodynamics (MHD) simulations of magnetic energy–powered explosion and find that for a point injection of $10^{61}$erg magnetic energy, $\eta_{B}$ retains a value of 15%(0.5 Mpc/$R_{B}$) at late stages, where $R_{B}$ is the bubble radius. The universal stellar mass density formed by a redshift of interest in units of the total baryonic density is denoted as $\eta_{*}$. With these variables and assuming that the radio jets send magnetic fields to large distances to fill a volume fraction of the universe, f, we obtain the mean magnetic field energy density

[3] $$\begin{array}{ll} \frac{B_{i}^{2}}{8\pi } & =3\times 10^{-4}\text{​}\left(\frac{\eta_{\text{M}}}{0.002}\right)\text{​}\left(\frac{\eta_{B}}{0.15}\right)\text{​}\left(\frac{\eta_{R}}{0.2}\right)\text{​}{\left(\frac{f}{0.2}\right)}^{-1}\eta_{*}\eta_{J}c^{2}\rho_{b}(z)\\ & =2.8\times 10^{-15}{\left(\text{μG}\right)}^{2}\left(\frac{\eta_{\text{M}}}{0.002}\right)\left(\frac{\eta_{B}}{0.15}\right)\left(\frac{\eta_{R}}{0.2}\right){\left(\frac{f}{0.2}\right)}^{-1}\\ & \times \left(\frac{\eta_{*}}{0.018}\right)\left(\frac{\eta_{J}}{0.05}\right){\left(\frac{1+z}{3}\right)}^{3}, \end{array}$$

where c is the speed of light, $\rho_{b}\left(z\right)$ the mean baryonic density at the redshift in question.

Let us now go through the numerical values of various variables, based solely on observational data. We adopt the universal stellar density of ($5.7\times 10^{7}\;M_{⊙}\;{\mathrm{Mpc}}^{-3}$, $3.6\times 10^{7}\;M_{⊙}\;{\mathrm{Mpc}}^{-3}$) (in comoving volume) at z=(2,3), respectively, from (28) converted to values corresponding to the Salpeter initial mass function, yielding $\eta_{*}=\left(0.018,0.011\right)$. From figure 10 of (29), we find radio loud fraction $\eta_{R}=\left(0.19,0.24\right)$ at z=(3,2), respectively; given the uncertainties involved, we shall adopt a single value of $\eta_{R}=0.2$, which is a good approximation for the entire redshift range of z= 0 to 4. The distribution of $\eta_{J}$ for the classic FR II sources (shown as a blue solid curve) is from (30). If f=1, we find $B_{i}$= 0.12 μG at z=2, which is the mean magnetic field strength over the entire universe.

**Fig. 2.**
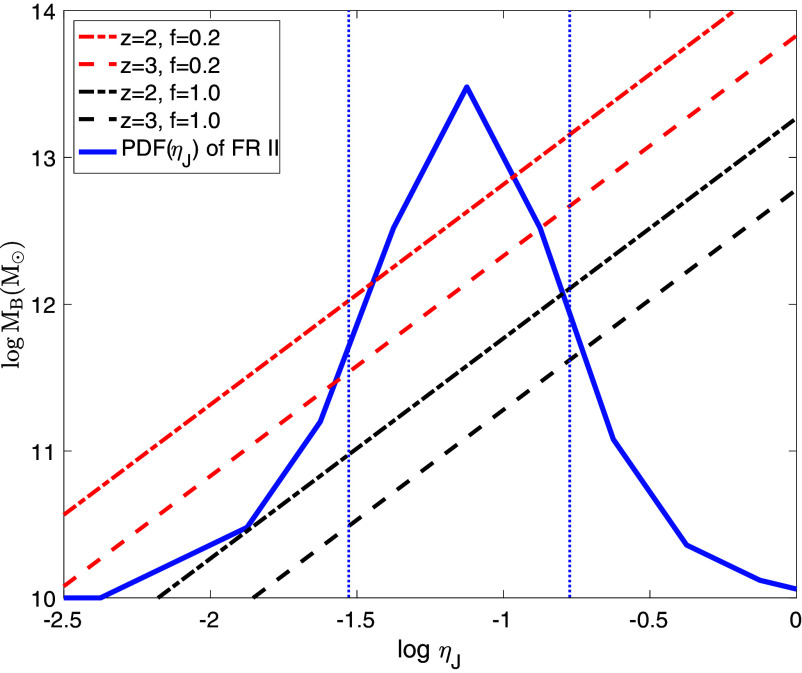
shows the magnetic Jeans mass, $M_{B}$, as a function of $\eta_{J}$, the fraction of the SMBH rest mass energy released in the form of powerful radio jets in radio-loud galaxies, at z=2 (dot-dashed lines) and z=3 (dashed lines). Two cases of filling factor f are shown: the two red lines are for f=0.2, whereas the two black lines are for f=1.0. Other relevant parameters are as follows. The observed mean stellar mass density of $5.7\times 10^{7}\;M_{⊙}\;{\mathrm{Mpc}}^{-3}$ and $3.6\times 10^{7}\;M_{⊙}\;{\mathrm{Mpc}}^{-3}$ at z=2 and z=3, respectively, are from (28). For the fraction of all relevant galaxies classified as radio loud galaxies, $\eta_{R}=0.2$ is adopted (29). The fraction of original jet magnetic energy remaining in the form of magnetic energy in the final giant radio bubbles is assumed to be $\eta_{B}=7.5\%$, estimated from simulations. Also shown as a solid blue curve are the observed probability density distributions of $\eta_{J}$ for the classic FR II sources (30).

We express the threshold halo mass $M_{B}$ due to magnetic pressure, as a function of $\eta_{J}$, shown in Fig. 2, where $\eta_{B}=0.075$ is adopted. The observed distribution of $\eta_{J}$ for the FR II sources shown as the blue solid curve has the ±1σ range indicated by the two vertical dotted blue lines. $M_{B}$ values at the two crossing points between an inclined line and the two vertical dotted blue lines indicate the ±1σ range of $M_{B}$ for that line. We find ±1σ range of $\left[2.2\times 10^{10},4.0\times 10^{11}\right]\;M_{⊙}$ and $\left[1.0\times 10^{11},1.3\times 10^{12}\right]\;M_{⊙}$ at z=3 and z=2, respectively, assuming f=1.0; ±1σ range becomes $\left[3.5\times 10^{11},4.5\times 10^{12}\right]\;M_{⊙}$ and $\left[1.1\times 10^{12},1.4\times 10^{13}\right]\;M_{⊙}$ at z=3 and z=2, respectively, if f=0.2.

## Discussion and Predictions

We have shown that, with a set of parameters anchored firmly on observational data, the magnetic pressure of accreting gas infused with magnetic fields originating from giant radio lobes exerts significant retarding effects on gas accretion into halos as massive as $10^{12\;\;\text{to}\;12.5}\;M_{⊙}$. Here, we discuss possible other effects and tests of this physical process.

### Effect of Parker Instability.

For a dark matter halo the density profile near the virial radius $r_{v}$ has a local slope α∼−2.3. Then, it can be shown that the minimum wavelength of the planar undular instability at the virial radius $r_{v}$ is $\lambda_{\min}=4\sqrt{2/(-1-\alpha )}\left(v_{A}/\sqrt{2}\sigma_{v}\right)r_{v}=5.0\left(v_{A}/\sqrt{2}\sigma_{v}\right)r_{v}$. Since $v_{A}/\sqrt{2}\sigma_{v}$ (Eq. **2**) is about unity or larger for the magnetic suppression to be significant, we see that the perturbation with the minimal wavelength, which is also the fastest growing mode, grows on a time scale of order five times the dynamic time of the halo at the virial radius. Hence, the induced Rayleigh-Taylor (RT) instability, if it exists, will unlikely be very important, since the relevant time scale close a halo is its dynamical time. In other words, if the gas eventually becomes unstable, the long waiting time of five dynamical times is already a significant retarding effect. Moreover, $\lambda_{\min}$ is much larger than $r_{v}$ and in fact larger than one-half of the circumference, such a planar undular instability then would not exist in the first place. Thus, we expect the RT instability due to magnetic buoyancy is unlikely to significantly alter the proposed magnetic pressure–based gas accretion suppression onto eligible halos.

### Magnetic Reconnection.

In our model, the initial magnetic field that we focus on is on large scales, of order 1 Mpc, larger than affected halos of virial radius of 300 kpc or smaller. Hence the magnetic field reaching a halo is on the scale of the halo size or larger. Given the normalized reconnection rate of order 0.1, inferred from observations and from theoretical calculations, it implies that the magnetic reconnection time is about ten times the system time (i.e., halo dynamical time), which is on the order of Hubble time at any redshift. Moreover, when the gas is en route to the halo, the reconnection time scale is still longer. This indicates that magnetic reconnection, which may take place to perhaps cancel some of the smaller scale stressed magnetic field on relatively shorter time scales, is unlikely to reduce significantly the overall magnetic energetics of gas endowed with large-scale magnetic field with $v_{A}=\sqrt{2}\sigma$ or larger.

### Volume Filling Fraction of Radio Bubbles.

We now return to the issue of volume filling fraction of the magnetic bubbles, f. We can only give a very rough estimate given uncertainties. Let us assume that the radio AGNs are dominated by massive galaxies, based on available observational evidence (e.g., ref. 30). To facilitate an estimation, let us assume that $0.5L_{*}$ galaxies and above are responsible for making the giant radio lobes; the mean separation of $0.5L_{*}$ galaxies is about 4 h^−1^ Mpc. For a Schechter function with a low-end slope of −1, one-half of the galaxy mass is contributed by galaxies above $0.5L_{*}$. Thus, if radio bubbles each have a mean radius of 0.5 Mpc for the galaxies considered, we have $f=2\times 4\pi {\left(0.5\;\mathrm{proper}\;\;\mathrm{Mpc}\right)}^{3}/3/{\left(4\;{\text{h}}^{-1}{\left(1+z\right)}^{-3}\;\mathrm{Mpc}\right)}^{3}∼14\%$ at =2, assuming that there is one occurrence of radio bubble event per $0.5L_{*}$ galaxy. Adopting the peak value of $\eta_{J}$ shown in Fig. 2, and assuming the average SMBH mass of the radio lobe launching AGNs is $10^{8}\;M_{⊙}$, corresponding to $L_{*}$ galaxies (31), we obtain the number of pairs of radio lobes per galaxy $N_{p}=0.9\;E_{60}^{-1}$, where $E_{60}=E_{R}/10^{60}$erg with $E_{R}$ being the mean energy of each radio lobe; the observed radio lobe total energy is in the range of a few times $10^{59}\;\;\text{to}\;10^{61}$erg (e.g., refs. 32 and 33). It thus seems that the overall volume filling fraction f may fall in the range of order 10 to 20%. In terms of galaxies, these regions around radio jet sources are likely highly biased. But the fraction of halos contained in these regions are likely substantially higher than f. Therefore, we expect that the suppression effect due to magnetic pressure of accretion onto relevant halos will be substantial. More importantly, the suppression effect will be spatially dependent, potentially giving rise to a different kind of modulation of galaxy formation across different environments.

### On the Global Downturn of Star Formation below *z* = 2 to 3.

The observation that this feedback effect strength due to magnetic pressure becomes strong enough by z=2−3 to have crossed a halo mass threshold of about $M_{B}∼10^{12}\;\;\text{to}\;10^{12.5}\;M_{⊙}$ is significant. This threshold halo mass is similar to the halo mass which separates cold and hot accretion modes (11). This implies that, while the halos more massive than about $M_{B}∼10^{12.5}\;M_{⊙}$ will be self-quenched due to lack of cooling, the smaller halos where cold accretion mode would otherwise operate are now hindered from accreting gas due to magnetic pressure. Consequently, star formation across the entire halo mass spectrum is now suppressed. This may be a major culprit causing the global downturn of star formation in the universe from z= 2 to 3 to the present.

At this point, it is perhaps useful to clarify one point. This external, preventive feedback proposed here is not exclusive and does not rule out any possible internal feedback from active galactic nuclei (AGN) or from supernovae, both of which are undoubtedly present and important. In fact, in galaxies with sufficient cooling flows, internal AGN feedback has been demonstrated by many authors (34–36) to be able to counter or delay cooling of the hot gaseous halo to significantly reduce star formation.

### On the Flattening of Schechter Luminosity Function below *z* = 2 to 3.

Another significant point to note is the rather prompt change in the observed slope of the galaxy rest-frame ultraviolet (UV) luminosity function around *z* = 2 to 3, from *α* = −1.94 at *z* = 2 to 3 to *α* = −1.56 at *z* = 1.0 to 1.6 (37). The traditional conjecture that supernovae exert negative feedback on low mass galaxies would be in stark contradiction to this observed change in galaxy luminosity function slope. The argument goes as follows. Star formation is the most vigorous at z> 2 to 3 (38). Therefore, if supernova feedback were responsible for suppressing star formation in progressively lower mass galaxies, the slope of the galaxy luminosity function at z> 2 to 3 would have been flatter than at lower redshift, when star formation is less vigorous and hence suppressing effect on low-mass galaxies relatively less severe. The negative effect due to magnetic pressure proposed here, however, provides a natural explanation in timing. Gas accretion suppression due to magnetic pressure becomes important only at z<2 or so and the suppression is increasingly more severe on smaller mass halos, leading to a flattening of the galaxy luminosity function at z<2. As to how flattened the slope is due to this effect can not be easily estimated without detailed cosmological simulations.

### An Argument for Preventive Feedback Processes.

Observations show a peak value of about 20% of the global baryon to total mass ratio around galaxies of halo mass ∼10^12^ in the Sloan Digital Sky Survey (SDSS) galaxy sample (e.g., ref. 39). This is tantalizing. This is perhaps a direct piece of evidence against interval feedback as being the primary actor for driving gas away, simply because there is not an amount of gas that corresponds to the global ratio to be driven away in the first place, at least at relatively low redshift. In other words, there is no widespread evidence of the existence of galaxies whose baryon to total mass ratios are close to what the global ratio would indicate. If our model bears the truth, one expects that such a “deficiency” of baryons should persist to z∼2 and then start to shift to more baryon-rich galaxies at higher redshift, but the shift will be halo mass dependent with lower mass halos remaining deficient longer. Presently, most observational data at high redshift are presented as the gas to stellar mass ratio as a function of galaxy stellar mass (e.g., ref. 40).

### Seeding Magnetic Field in Galaxies and Damped Lyman Alpha Systems.

If a significant volume of the intergalactic space can attain a magnetic field of a strength of 0.1 μG or so, observed large magnetic field in some damped Lyman alpha systems at moderate redshift may be accounted for; for example, a simple contraction from the mean intergalactic gas density of ∼10^−5^ cm^−3^ to a typical interstellar medium density of 1 cm^−3^ would amplify a 0.1 μG field to an amplitude of 200 μG, readily explaining an amplitude of 84 μG in a galaxy at z=0.7 (41). Simulations have shown that damped Lyman alpha systems at moderate redshift of z= 2 to 3 are largely caused by circumgalactic filaments and sheets with a typical volumetric density in the range of $10^{-3}\;\;\text{to}\;10^{-1}\;\;{\text{cm}}^{-3}$ (42). Contraction alone from the mean intergalactic gas density of ∼10^−5^ cm^−3^ would result in a field in the range 2 to 50 μG for damped Lyman-alpha systems (DLAs), consistent with the observed values of typically a few microgauss (43).

### Magnetic Field in the Warm-Hot Intergalactic Medium (WHIM) and Clusters.

In the preceding subsection, we discuss the overall magnetic field in the IGM that a Faraday rotation measure based on line-of-sight integrals may yield. An estimate of the magnetic field in filaments, i.e., the WHIM at zero redshift (9). which make up a large portion of the missing baryons, may be made. Assuming that the magnetic field has largely been seeded due to the peak AGN activities at z=2, then the magnetic field in filaments of density $n_{e}$ at z=0 is

[4] $$\begin{array}{c} \begin{array}{c} B_{\mathrm{WHIM}}\left(n_{e}\right)=0.66\;\mu G\left(\frac{\overset{¯}{B}}{0.1\;\mu G}\right){\left(\frac{n_{e}}{10^{-4}\;\;{\text{cm}}^{-3}}\right)}^{2/3}, \end{array} \end{array}$$

assuming no further dynamo or other amplification. With a 0.1 to 1 μG magnetic field expected in WHIM filaments, if converging shocks compressing and forming filaments can accelerate or re-energize electrons, filaments may be detected in synchrotron radiation by the next-generation radio facilities, such as Square Kilometer Array (SKA). Current observations have already begun to detect such emission from filaments even assuming magnetic fields weaker than our estimates (e.g., ref. 44), The expected field strength is roughly consistent with synchrotron radiation observations, estimated based on energy equipartition assumption, of the magnetic field in local filaments (e.g., ref. 45).

In the cores of clusters of density $n_{e}∼10^{-3}\;\;\text{to}\;10^{-2}\;\;{\text{cm}}^{-3}$ a field strength of several microgauss or tens of microgauss is expected, even in the absence of any further amplification. This is in line with observations (e.g., ref. 46). What is perhaps more significant to note is that, if the cluster center is anchored by a field strength of several microgauss or tens of microgauss, a field strength of several 0.1 μG to several microgauss may exist in the outskirts of clusters of galaxies in our model, which appears to be observed (e.g., refs. 46 and 47).

### Cross-Correlations between Faraday Rotation Measure and Others.

In our model, we expect that magnetic field injected into the IGM is most concentrated in protoclusters. As such, a significant cross-correlation signal is expected between protoclusters and Faraday rotation measure (RM) along a same line of sight. To enhance the signal of this measure, one may denominate the cross-correlation between protoclusters and RM along the lines of sight through protocluster by the cross-correlation between protoclusters and RM along random lines of sight. This may allow one to pick out the line-of-sight magnetic field strength in the protoclusters fairly easily.

In addition to cross-correlating protoclusters with Faraday RM to pick out the magnetic field strength in the protoclusters, one may also compute the cross-correlation between Lyman alpha forest transmitted flux and RM. The premise behind this method is that the Lyman forest transmission in protoclusters of galaxies is substantially different from the Lyman forest transmission in typical, average lines of sights. This method shall yield RM measure as a function of Lyman alpha transmission flux. As long as there is a difference in Lyman alpha transmission flux between protocluster regions and nonprotocluster regions, one may be able to teased out RM in protoclusters in the future facilitated by SKA observations.

### Deposition of Thermal Energy in IGM Due to Expanding Lobe Shock Heating.

**Fig. 3.**
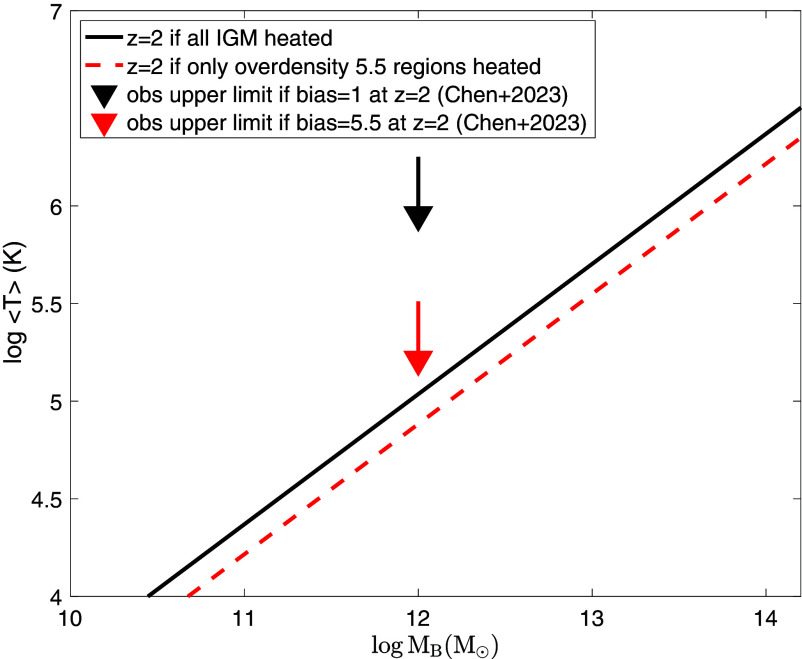
shows the expected mean temperature per baryon in the entire universe as a function of the magnetic Jeans mass $M_{B}$ at redshift z=2, for two cases, one assuming that the entire IGM is heated (black solid line) and the other assuming that only regions with overdensity of 5.5 are heated (red dashed line). Also shown are *Upper* limits observationally derived at z=2 (48) for two cases, one assuming that the entire IGM is heated with a bias factor equal to unity (blue arrow), and the other assuming that only regions that have turned around from universal expansion are heated with a bias factor equal to 5.5 (red arrow). In shock propagation, zero metallicity for the ambient gas is assumed.

The amount of thermal energy deposited in the IGM or protocluster regions or wherever can be calculated. We consider two regimes. If the cooling time of the heated postshock regions due to the expanding lobe is longer than the Hubble time at the redshift in question, then all thermalized energy is counted. On the other hand, if the cooling time of the heated postshock regions is shorter than the Hubble time, the apparent amount of all thermalized energy will be reduced by the ratio of the former to the latter, when one does a time averaging. Assuming zero metallicity for the ambient gas into which the radio lobe–driven shocks are propagating, the results are shown in Fig. 3, where the amount of energy shown in the y axis is expressed as the mean temperature of the IGM as a function of the magnetic Jeans mass $M_{B}$. Two cases are shown, one assuming that the entire IGM is heated with a bias factor equal to unity (black solid line), and the other assuming that only regions that have turned around from universal expansion are heated with a bias factor equal to 5.5 (red dashed line). In the latter case, the mean temperature is still averaged over all gas in the universe. Also shown are upper limits observationally derived at z=2 48 for two cases, one assuming that the entire IGM is heated with a bias factor equal to unity (blue arrow), the other assuming that only regions that have turned around from universal expansion are heated with a bias factor equal to 5.5 (red arrow), because the observationally derived upper limits depend on the bias factor of the heated region. We see that, so long as $M_{B}\leq 10^{12}\;\;\text{to}\;10^{12.5}\;M_{⊙}$, the thermal energy injected by expanding giant radio lobes is consistent with current observational limit.

### Magnetic Field at Virial Radius As a function of Halo Mass.

For halos less massive than the magnetic Jeans mass $M_{B}$, we expect that accreting gas will be prevented from entering the virial radius. As a result, the field may accumulate outside the virial radius of such a halo. Consequently, we expect that the magnetic field strength at the virial radius (assuming overdensity of 100) will be just at the threshold level for each halo at lower redshift at

[5] $$\begin{array}{c} \begin{array}{c} B_{h}\left(\sigma_{v}\right)=\sqrt{600\;\pi \Omega_{B}\rho_{c,0}{\left(1+z\right)}^{3}}\sigma_{v}, \end{array} \end{array}$$

where $\sigma_{v}$ is the 1-d velocity dispersion of the halo, $\rho_{c}$ is the critical density of the universe at z=0, and $\Omega_{B}$ baryon density. This prediction is potentially testable by SKA in the future.

### Suggestion on How to Implement This MHD Effect.

A simple version of how to implement this MHD effect may be described as follows, although more sophisticated implementation may be devised. First, one runs radio jet expansion simulation not in a cosmological simulation box but in an isolated box beforehand to produce a standard mold of magnetic field structures in a bubble of size 1 proper Mpc, to be inserted into the cosmological simulation. One may inject a prescribed amount of magnetic energy into the center over 100 Myr to mimic FR II sources. Second, one runs a cosmological MHD simulation of a sufficiently large box to contain one cluster of galaxies by z=0. One then retraces back to the starting redshift to identify a region that contains the protocluster of the cluster found at z=0. Then, one reruns the simulation with static meshrefinement in the zoom-in region that contains the protocluster from the starting redshift. At z=2, one adds a pair of radio bubbles of radius 1 proper Mpc for each of the massive galaxies at a distance of 1 Mpc from that galaxy in the protocluster using the mold in the first step with appropriate adjustments in velocity and density field based on the local values in the cosmological simulation box and then restarts the simulation from z=2 to run to z=0.

## Conclusions

Utilizing a set of parameters fully anchored on observational data, we show that giant radio lobes from supermassive black holes residing in massive galaxies can inject enough magnetic energy so as to exert a major negative feedback effect, by significantly retarding or preventing gas accretion onto halos as massive as $M_{B}=10^{13}\;M_{⊙}$ by z=2, depending on the volume filling fraction of the radio bubbles in the universe, thanks to the magnetic pressure of the accreting gas. The accretion suppression effect increases with decreasing halo mass. We shall call $M_{B}$ the magnetic Jeans mass.

The implication is profound. That is, by z= 2 to 3, halos where cold accretion mode would otherwise operate are now significantly prevented from accreting gas due to magnetic pressure. This negative feedback process may be the culprit for causing the global downturn in star formation in the universe from z= 2 to 3 to the present. This feedback mechanism also provides a natural explanation for the rather prompt change in the slope of the galaxy rest-frame UV luminosity function around *z* = 2 to 3, from α = −1.94 at *z* = 2.2 to 3 to *α* = −1.56 at *z* = 1.0 to 1.6 (37).

Finally, a number of ramifications and predictions due to this process are given. First, magnetic fields for a host of systems, such as galaxies and damped Lyman alpha systems at moderate redshift, and extragalactic filaments and clusters of galaxies low redshift, may be accounted for. Second, the thermal energy injected by shocks due to supersonically expanding magnetic bubbles is significant but in agreement with current observational upper limits. Third, cross-correlations between protoclusters and Faraday rotation measures should be able to test the predicted magnetic field directly. Cross-correlations between Faraday rotation measure and Lyman alpha forest flux spectrum can provide additional information on this.

## Materials and Methods

A combination of empirical observational data and bubble evolution analytics is used to quantify a potentially significant preventive effect on the dynamics of intergalactic gas accretion onto dark matter halos. We make use of the observed abundance of radio galaxies, the observed fractional energy released in the form of large radio jets in terms of supermassive black rest mass energy and the total amount of mass in supermassive black holes in units of the stellar mass formed in the universe. We deduce the total amount of magnetic energy in giant radio lobes based on these observational data, in combination with results obtained based on MHD radio bubble expansion simulations that estimate the surviving magnetic energy when realistic bubbles reach a radius of order 1 Mpc. Combining these results, we estimate the magnetic pressure of gas accreting into dark matter halos and show that it could provide a significant hinderance for gas accretion, thus potentially preventing or slowing or retarding gas accretion onto dark matter halos as massive as $10^{12}\;M_{⊙}$ at z= 2 to 3. Uncertainties and spatial variations of this effect are discussed, depending on the volume filling factor of the radio bubbles.

## Data Availability

There are no data underlying this work.
